# Combined transplantation of human mesenchymal stem cells and human retinal progenitor cells into the subretinal space of RCS rats

**DOI:** 10.1038/s41598-017-00241-5

**Published:** 2017-03-15

**Authors:** Linghui Qu, Lixiong Gao, Haiwei Xu, Ping Duan, Yuxiao Zeng, Yong Liu, Zheng Qin Yin

**Affiliations:** 1 Southwest Hospital, Southwest Eye Hospital, Third Military Medical University, Chongqing, 400038 China; 2 Key Lab of Visual Damage and Regeneration & Restoration of Chongqing, Chongqing, 400038 China

## Abstract

Retinitis pigmentosa (RP) is one of hereditary retinal diseases characterized by the loss of photoreceptors. Cell transplantation has been clinically applied to treat RP patients. Human retinal progenitor cells (HRPCs) and human bone marrow-derived mesenchymal stem cells (HBMSCs) are the two commonly and practically used stem cells for transplantation. Since combined transplantation could be a promising way to integrate the advantages of both stem cell types, we transplanted HRPCs and HBMSCs into the subretinal space (SRS) of Royal College of Surgeons (RCS) rats. We report that HRPCs/HBMSCs combined transplantation maintains the electroretinogram results much better than HRPCs or HBMSCs single transplantations. The thickness of outer nuclear layer also presented a better outcome in the combined transplantation. Importantly, grafted cells in the combination migrated better, both longitudinally and latitudinally, than single transplantation. The photoreceptor differentiation of grafted cells in the retina of RCS rats receiving combined transplantation also showed a higher ratio than single transplantation. Finally, activation of microglia and the gliosis of Müller cells were more effectively suppressed in combined transplantation, indicating better immunomodulatory and anti-gliosis effects. Taken together, combining the transplantation of HRPCs and HBMSCs is a more effective strategy in stem cell-based therapy for retinal degenerative diseases.

## Introduction

Retinitis pigmentosa (RP) is a set of hereditary retinal diseases characterized by the degeneration of rod and cone photoreceptors^1^. Approximately 1 in 4000 people are affected by RP, and their symptoms are highly variable^2^. Dysfunction of rod photoreceptors precede that of cone receptors, bringing early night blindness to RP patients^3^. The subsequent severe rod and cone photoreceptor death leads to progressive visual field losses and usually causes blindness by age 60^1, 3^. A specific medicine to cure RP has not yet been developed. Vitamin A, docosahexaenoic acid and valproic acid were found to slow the visual function loss and show positive effects in RP patients^4–6^. Other pharmacological treatments including neurotrophic factors and anti-inflammatory factors are still on their way to being used to treat patients^7–9^. Potential cell transplantation is now becoming a promising way to rescue dead cells and reform neural connections in animal models^10–14^.

Since the retina can be viewed directly from the pupil, in addition to its immune privilege characteristic, the retina has become an ideal site for cell transplantation^15^. In general, potential cells for retinal transplantation should have the following features: the ability to be easily cultured *in vitro*, the ability to migrate, the ability to differentiate into typical retinal cells and the ability to integrate into the retina^16^. According to this principle, the types of potential cells could be embryonic stem cells, human bone marrow derived mesenchymal stem cells (HBMSCs), neural stem cells, human retinal progenitor cells (HRPCs), olfactory unsheathing cells, Müller cells and adult photoreceptor or retina pigment cells^17–22^. All of these cells, which come from blastocysts, bone marrow, umbilical cord blood, adult forebrain or hippocampus, embryonic or neonatal retina, olfactory bulb and adult retina, have been used in transplanting potential cells into the subretinal space (SRS)^16^. Considering the compatibility, functions of proliferation, differentiation and integration of potential cells, HRPCs appear to be a perfect candidate for transplantation.

Transplantation of HRPCs into the SRS of animal models including Royal College of Surgeons (RCS) rats and rd1 mice has been performed by several groups^23, 24^. In both studies, visual function was preserved after cell transplantation, which was characterized by increases in the amplitude of electroretinograms (ERG) and the thickness of outer nuclear layer (ONL). However, the protective effect of HRPCs transplantation is barely satisfactory. In addition, the differentiation of transplanted cells was hardly observed. HBMSCs were also used in SRS transplantation to treat retinal degenerative diseases. After transplantation, visual function could be improved by reducing the apoptosis of photoreceptors and increasing the electrophysiological response^25–28^. HBMSCs were believed to produce cytokines and neurotrophic factors, which were able to improve the living conditions of retinal cells and activate the resident stem cells within retina^29–31^. However, the differentiation of HBMSCs into retinal cells was critically difficult, indicating that the improvement of visual function has an apparent limit.

Combined transplantation appears to be an efficient way to gather the unique features of different stem cells. So far, many studies have been performed using combined transplantation of two different stem cells to treat diseases such as spinal cord injury, autism, diabetes, myocardial infarction and ischemia^32–37^. The results of these combined transplantation therapies presented good outcomes and no obvious side effects, indicating that combined transplantation is a safe and effective method. Taken together, combined transplantation of HRPCs and HBMSCs in retinal degenerative disease appears to be a possible way to integrate their advantages that has yet to be reported.

Therefore, in the present study, HRPCs and HBMSCs were transplanted either singly or combined into the SRS of RCS rats. Using ERG, immunofluorescence, western-blot and RT-PCR, we assayed the efficacy of combined transplantation of these two cells.

## Results

### Identification of HRPCs

**Figure 1:**
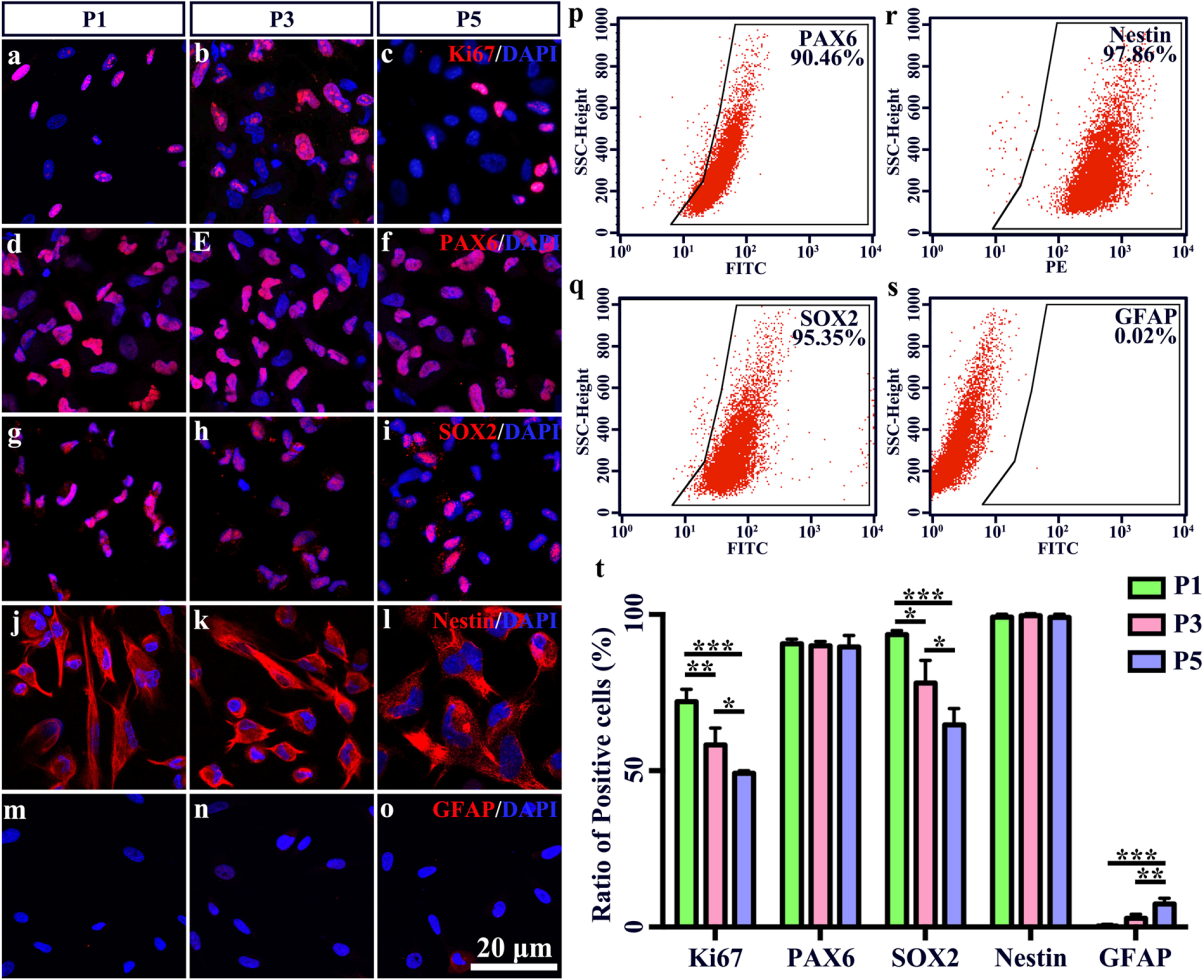
Identification of human retinal progenitor cells (HRPCs). (**a**–**o**) Five markers including Ki67, Pax6, Sox2, Nestin and GFAP were used to identify the characteristics of HRPCs in passages1, 3 and 5. (**p**–**s**) Flow cytometry analysis of HRPCs with Pax6, Sox2, Nestin and GFAP. (**t**) Corresponding statistic analysis of (**a**–**o**). For Ki67 staining, the significance between P1 and P3 is P = 0.004637. The significance between P1 and P5 is P = 0.000349. The significance between P3 and P5 is P = 0.028506. For SOX2 staining, the significance between P1 and P3 is P = 0.010751. The significance between P1 and P5 is P = 0.000505. The significance between P3 and P5 is P = 0.020324. For GFAP staining, the significance between P1 and P5 is P = 0.000635. The significance between P3 and P5 is P = 0.005400. *P < 0.05; **P < 0.01; ***P < 0.001.

As the main stem cell population in the retina, RPCs should display basic progenitor characteristics including proliferation and stemness. To verify that the cells that we harvested from fetal eye are HRPCs, we tested for the expression of Ki67 (proliferation marker), PAX6 and SOX2 (retinal progenitor marker), and Nestin (retinal stem cell marker) via immunocytochemistry (see supplementary methods). Our results showed that the ratio of Ki67-positive proliferating cells significantly decreased during passaging (72.13% ± 3.17% at passage 1, 58.27% ± 4.42% at passage 2 and 49.20% ± 0.65% at passage 3) (Fig. 1a–c,t). At same time, the ratio of SOX2-positive stem cells also significantly decreased during passaging (93.57% ± 1.02% at passage 1, 78.03% ± 5.94% at passage 2 and 64.7% ± 4.25% at passage 3) (Fig. 1g–i,t). On the other hand, the ratio of PAX6- and Nestin-positive progenitor cells remained stable during passaging (90.63% ± 1.02% and 99.17% ± 0.74% at passage 1, 89.93% ± 1.16% and 99.60% ± 0.50% at passage 2 and 89.57% ± 3.00% and 99.01% ± 0.82% at passage 3) (Fig. 1d–f,j–l,t). Besides, Crx, NeuroD1, Tuj1, Vimentin and Nrl showed a stable expression ratio during passaging (Supplementary Fig. 1a–o,s). But the expression of Otx2 decreased during passaging (Supplementary Fig. 1p–r,s). However, the ratios of the glial marker remained at low levels (less than 8%) but showed a non-significant increase during passaging (0.33% ± 0.47% at passage 1, 2.77% ± 1.06% at passage 2 and 7.37% ± 1.47% at passage 3) (Fig. 1m–o,t). FACS was used to confirm the expression level of biomarkers. The results showed that, on passage 3, the positive percentages of PAX6, Nestin, SOX2 and GFAP were 90.46%, 97.86%, 95.35% and 0.02%, respectively (Fig. 1p–s), which was consistent with the immunofluorescence results.

### Identification of HMSCs

**Figure 2:**
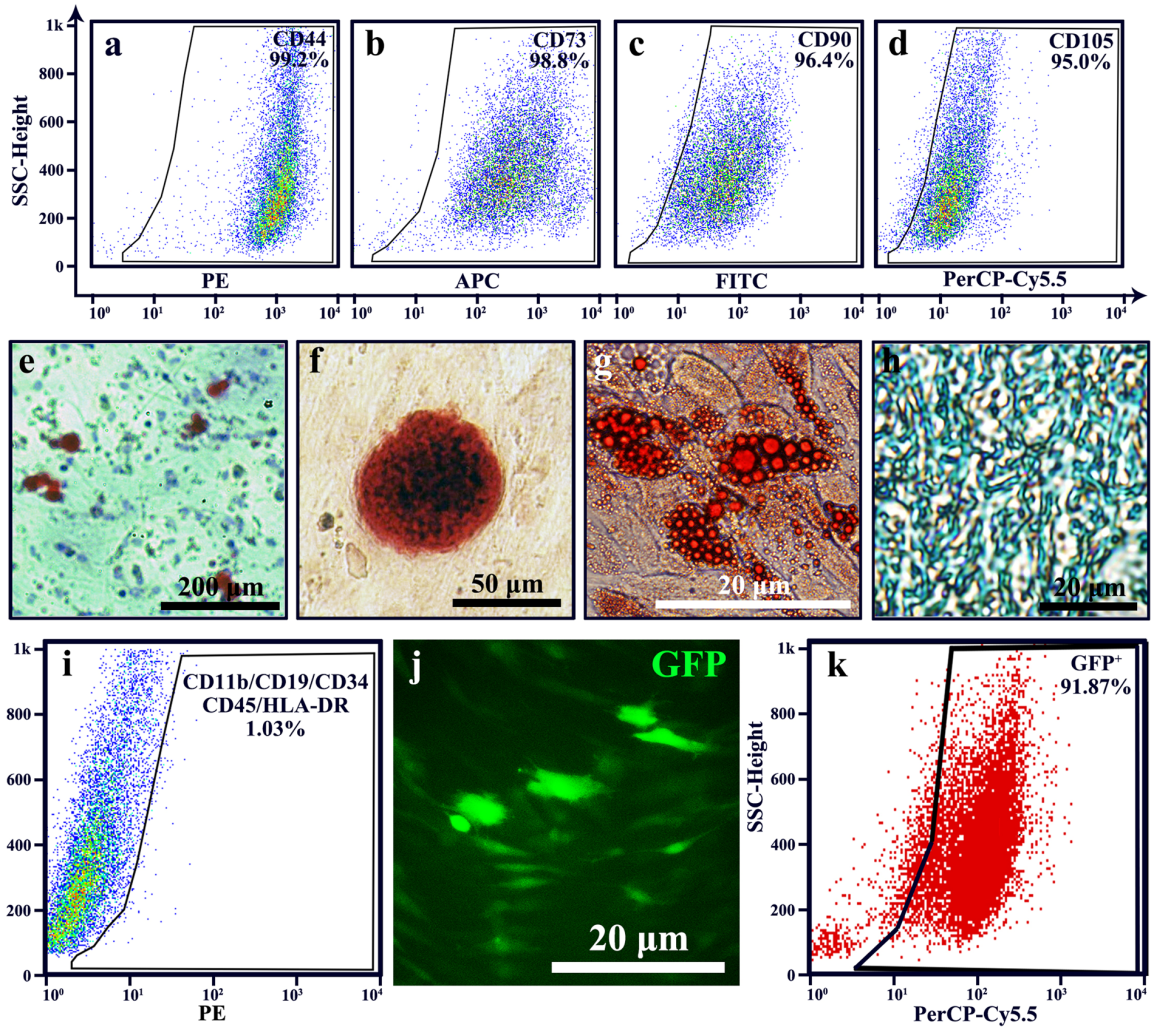
Identification of human bone mesenchymal stem cells (HBMSCs). (**a**–**d**) Flow cytometry analysis of HBMSCs with CD44, CD73, CD90 and CD105. (**e**–**h**) Characteristics of HBMSCs. (**e**,**f**) Bone-forming test, results showed that there were Alizarin Red S staning mineralized matrixes formed. (**g**) Adipocyte-differentiating test showed that lipid vesicles were visualized with Oil Red O. (**h**) Cartilage-forming test showed that Alcian Blue staining cartilage could be visualized. (**i**) Flow cytometry analysis of negative marker cocktail. Results showed that CD11b, CD19, CD34, CD43 and HLA-DR were negative in the HBMSCs. (**j**,**k**) Identification of GFP-lentivirus transfected HBMSCs. (**k**) Image of GFP showed that more than 80% cells were GFP positive. (**j**) Flow cytometry analysis of HBMSCs showed that over 90% cells were positive for GFP.

HBMSCs can express CD44, CD73, CD90 and CD105 simultaneously. Using FACS, we found that the positive percentages of CD44, CD73, CD90 and CD105 in HBMSCs on passage 3 were 99.2%, 98.8%, 96.4% and 95%, respectively (Fig. 2a–d) (see supplementary methods). HBMSCs should also be able to show multi-lineage potential^38^. After being induced in osteogenic conditions for 10–12 days, HBMSCs formed sphere- or olive-matrices (Fig. 2e). With the staining of Alizarin Red S, osteogenic calcium depositions could be visualized (Fig. 2f). An adipocyte-differentiation test showed that HBMSCs were able to form vesicle-like structures. After staining with Oil Red O, lipid vesicles could be visualized (Fig. 2g). To test whether our HBMSCs had cartilage-forming ability, HBMSCs were incubated with chondrogenic medium for 4 weeks. With Alcian Blue staining, cartilage could be detected (Fig. 2h).

To exclude the possibility of contamination with other cells, CD11b, CD19, CD34, CD43 and HLA-DR expression was tested by FACS, with negative results (Fig. 2i). All these results illustrate that the isolated cells from bone marrow were HBMSCs. To perform transplantation surgery, HBMSCs were transfected with lentivirus-GFP. Using immunofluorescence and FACS, approximately 90% of HBMSCs were GFP positive cells (Fig. 2j,k).

### ERG recording

**Figure 3:**
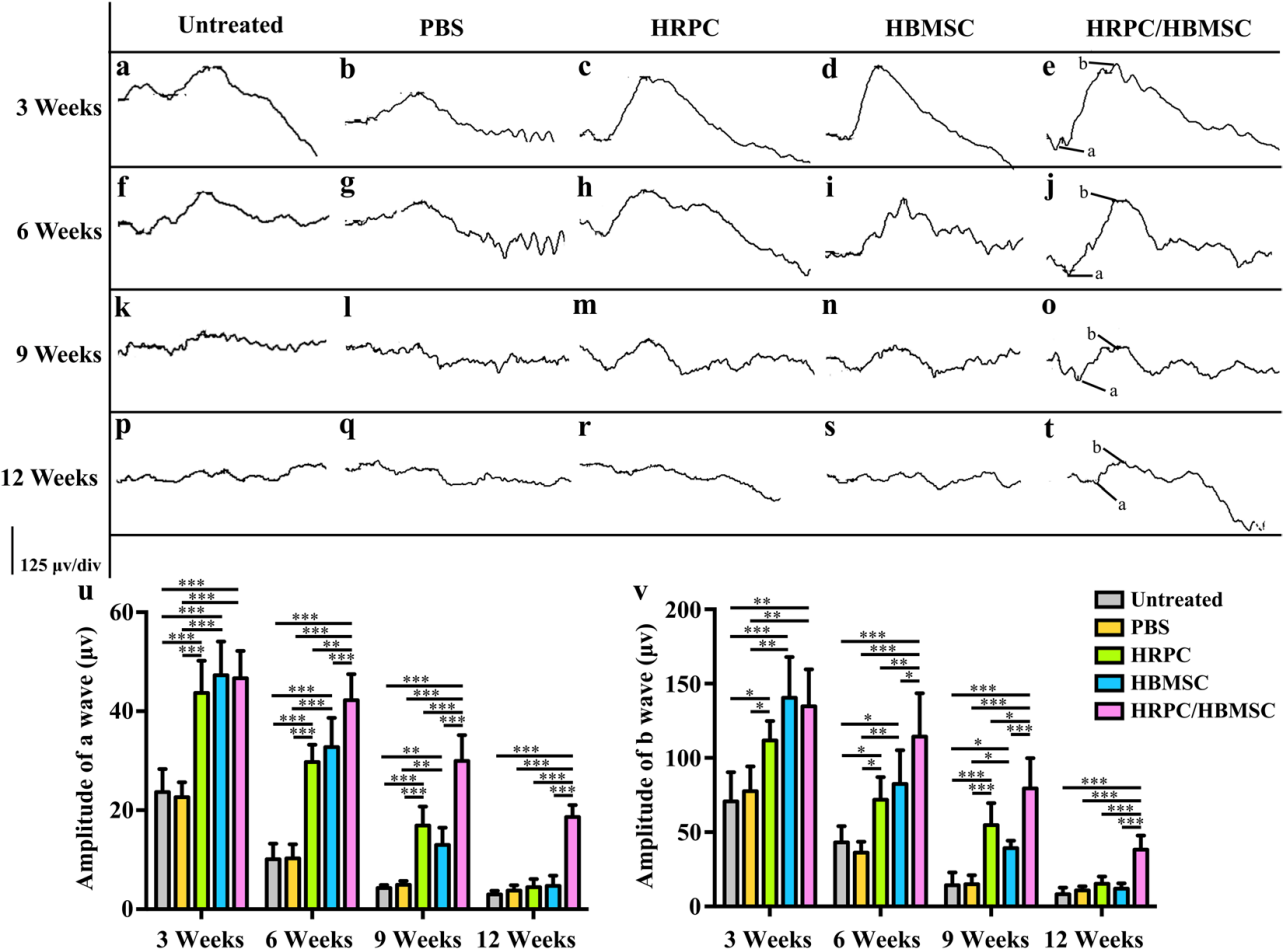
Comparison of electroretinograms (ERG) test after single and combined cell transplantation into subretinal space (SRS). (**a**,**f**,**k**,**p**) Representative ERG test of untreated RCS rats on 3, 6, 9 and 12 weeks (the same time point with operational groups). (**b**,**g**,**l**,**q**) Representative ERG test after PBS injection on 3, 6, 9 and 12 post operational weeks. (**c**,**h**,**m**,**r**) Representative ERG test after human retinal progenitor cells (HRPCs) injection on 3, 6, 9 and 12 post operational weeks. (**d**,**i**,**n**,**s**) Representative ERG test after human bone mesenchymal stem cells (HBMSCs) injection on 3, 6, 9 and 12 post operational weeks. (**e**,**j**,**o**,**t**) Representative ERG test after HRPCs and HBMSCs double injection on 3, 6, 9 and 12 post operational weeks. (**u**) Statistical analysis of the amplitude of ERG a wave in all 5 groups at 4 time points. (**v**) Statistical analysis of the amplitude of ERG b wave in all 5 groups at 4 time points. Results showed that all cell transplantation groups displayed increases of the ERG amplitude. Combined transplantation showed longest vision-functional rescue effect. *P < 0.05; **P < 0.01; ***P < 0.001. (n = 3).

As an electrophysiology-testing method, ERG can reflect the function of the retina, especially the function of photoreceptors by measuring the amplitudes of both a and b waves. The main purpose of transplantation into RCS rats is to measure the functional improvement of injured photoreceptors. Thus, ERGs were recorded on all post-operational rats at 3, 6, 9 and 12 weeks after cell transplantations. The results showed that both combined transplantation or single transplantation displayed a significant improvement of the amplitude of a and b waves at 3, 6 and 9 weeks, compared to those in the untreated group and PBS injection group (Fig. 3a–t,u,v). In particular, there was no significant difference among single HRPCs transplantation, single HBMSCs transplantation or combined transplantation by 3 weeks (Fig. 3a–e,u,v). However, the amplitude of both a and b waves in the combined transplantation group (a wave: 42.25 ± 4.52, b wave: 114.33 ± 25.23) increased significantly by 6 weeks, compared to those in the single HRPCs transplantation group (a wave: 29.75 ± 3.03, P = 0.000910; b wave: 71.93 ± 13.18, P = 0.006055) and single HBMSCs transplantation group (a wave: 32.78 ± 5.10, P = 0.006987; b wave: 82.55 ± 19.58, P = 0.030260) (Fig. 3f–j,u,v). By 9 weeks, though decreased, the amplitudes of both a and b waves in the combined transplantation group (a wave: 29.95 ± 4.52; b wave: 79.40 ± 17.74) were significantly higher than that in the single HRPCs transplantation group (a wave: 16.93 ± 3.30, P = 0.000054; b wave: 54.75 ± 12.84, P = 0.013526) and single HBMSCs transplantation group (a wave: 13.00 ± 3.00, P = 0.000003; b wave: 39.25 ± 4.40, P = 0.000378) (Fig. 3k–o,u,v). However, after 12 weeks, there was no significant difference of ERG readings among single HRPCs transplantation, single HBMSCs transplantation and PBS injection groups. Meanwhile, the amplitudes of a and b waves in the combined transplantation group (a wave: 18.63 ± 2.09; b wave: 38.23 ± 8.20) still showed significantly better effects over the single HRPCs transplantation group (a wave: 4.45 ± 1.41, P = 0.000000; b wave: 15.23 ± 4.29, P = 0.000032) and the single HBMSCs transplantation group (a wave: 4.73 ± 1.79, P = 0.000000; b wave: 11.93 ± 3.23, P = 0.000007) (Fig. 3p–t,u,v). There showed now significant difference between untreated group and PBS injection group in all time points (Fig. 3a,b,f,g,k,l,p,q,u,v).

### ONL thickness analysis

**Figure 4:**
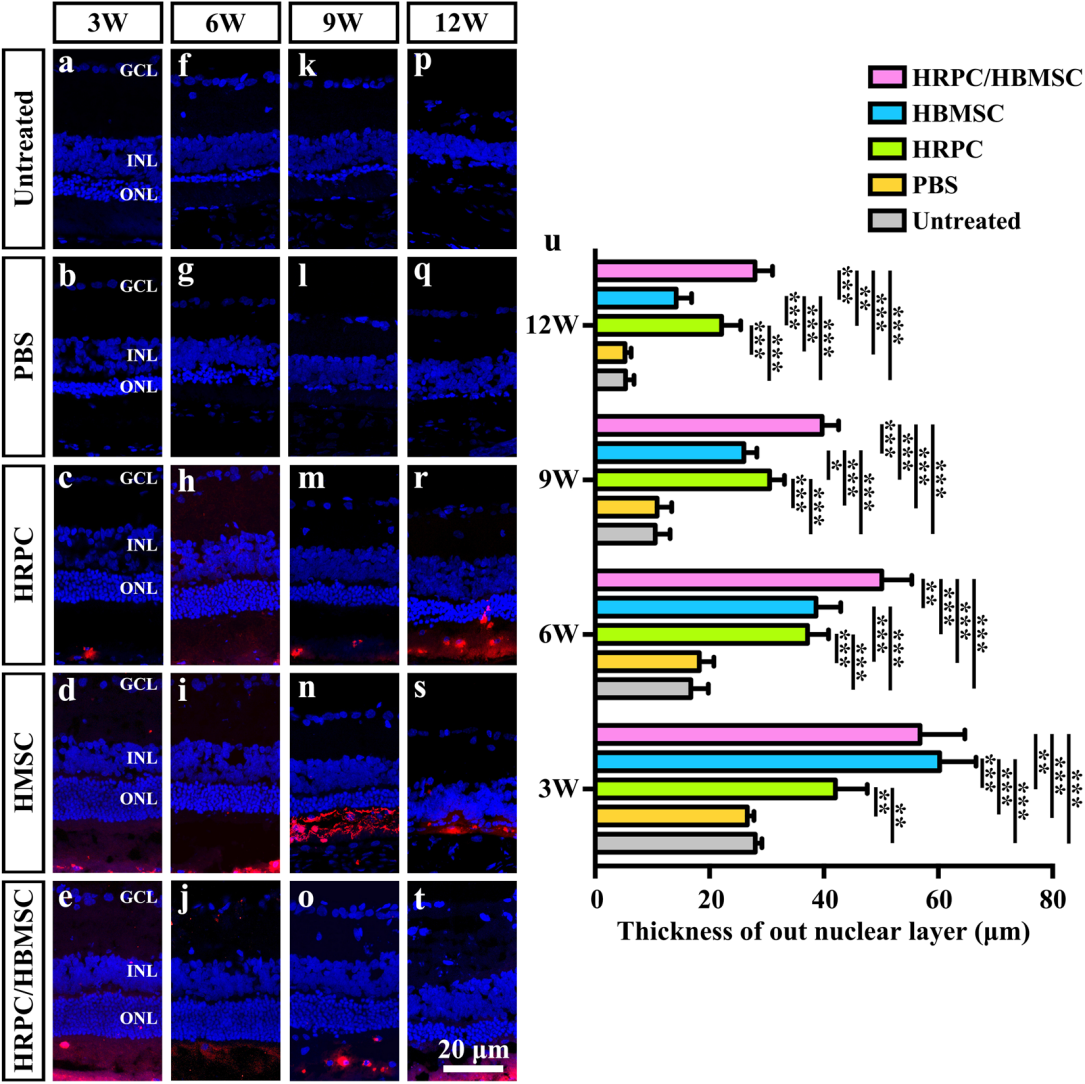
Comparison of out nucleus layer (ONL) thickness after single and combined cell transplantation into subretinal space (SRS). (**a**,**f**,**k**,**p**) Representative ONL thickness of untreated RCS rats on 3, 6, 9 and 12 weeks (the same time point with operational groups). (**b**,**g**,**l**,**q**) Representative image of ONL thickness after PBS injection on 3, 6, 9 and 12 post operational weeks. (**c**,**h**,**m**,**r**) Representative ONL thickness after human retinal progenitor cells (HRPCs) injection on 3, 6, 9 and 12 post operational weeks. (**d**,**i**,**n**,**s**) Representative ONL thickness after human bone mesenchymal stem cells (HBMSCs) injection on 3, 6, 9 and 12 post operational weeks. (**e**,**j**,**o**,**t**) Representative ONL thickness after HRPCs and HBMSCs double injection on 3, 6, 9 and 12 post operational weeks. (**q**) Corresponding statistical analysis of the ONL thickness in all 5 groups at 4 time points. Results showed that all cell transplantation groups displayed increases of the ONL thickness, which indicated the protection of photoreceptor. Combined transplantation showed longest photoreceptor-protection effect. *P < 0.05; **P < 0.01; ***P < 0.001. (n = 3).

Since retinal function was profoundly improved after combined transplantation, we also investigated the morphological evidence, which supported our findings. RCS rats mainly suffered from loss of photoreceptors. Therefore, the ONL thickness was measured to assay for the protection of the ONL among different types of cell transplantations. The results showed that all cell transplantation groups presented significantly thicker ONLs than the untreated group and PBS-alone injection groups at 3, 6, 9 and 12 weeks post-operation (Fig. 4a–p,q). The combined transplantation groups displayed a significant increase of ONL thickness (56.82 ± 6.79) over the HRPCs transplantation group (42.02 ± 4.81, P = 0.001122) by 3 weeks post-operation (Fig. 4a–e,u). In addition, no significant differences were observed between the combined transplantation (56.82 ± 6.79) and HBMSCs transplantation groups (60.23 ± 5.53, P = 0.369468) at this time point (Fig. 4a–e,u). By 6 and 9 weeks post-operation, the ONL thickness in the combined transplantation group showed a significant increase (6 weeks: 50.12 ± 4.55; 9 weeks: 39.68 ± 2.49), compared to that in the HRPCs (6 weeks: 37.15 ± 3.18, P = 0.000273; 9 weeks: 30.51 ± 2.24, P = 0.000154) and HBMSCs (6 weeks: 38.61 ± 3.74, P = 0.000791; 9 weeks: 26.03 ± 1.97, P = 0.000002) transplantation groups (Fig. 4f–o,u). However, the ONL thickness in the HBMSCs transplantation group significantly decreased by 9 weeks (26.03 ± 1.97), compared to that in the HRPCs transplantation group (30.51 ± 2.24, P = 0.026731) (Fig. 4k–o,u), which increased by 12 weeks post-operation (HBMSCs: 14.17 ± 2.34; HRPCs: 22.13 ± 2.86, P = 0.000392) (Fig. 4r,s,u). At that time, the combined transplantation groups (27.95 ± 2.64) still maintained a significant increase of ONL thickness over the HRPCs (22.13 ± 2.86, P = 0.004702) and HBMSCs (14.17 ± 2.34, P = 0.000001) transplantation groups (Fig. 4p–t,u). There showed now significant difference on ONL thickness between untreated group and PBS injection group in all time points, which is corresponding to the ERG results (Fig. 4a,b,f,g,k,l,p,q,u).

### Longitudinal and latitudinal migration of transplanted cells

**Figure 5:**
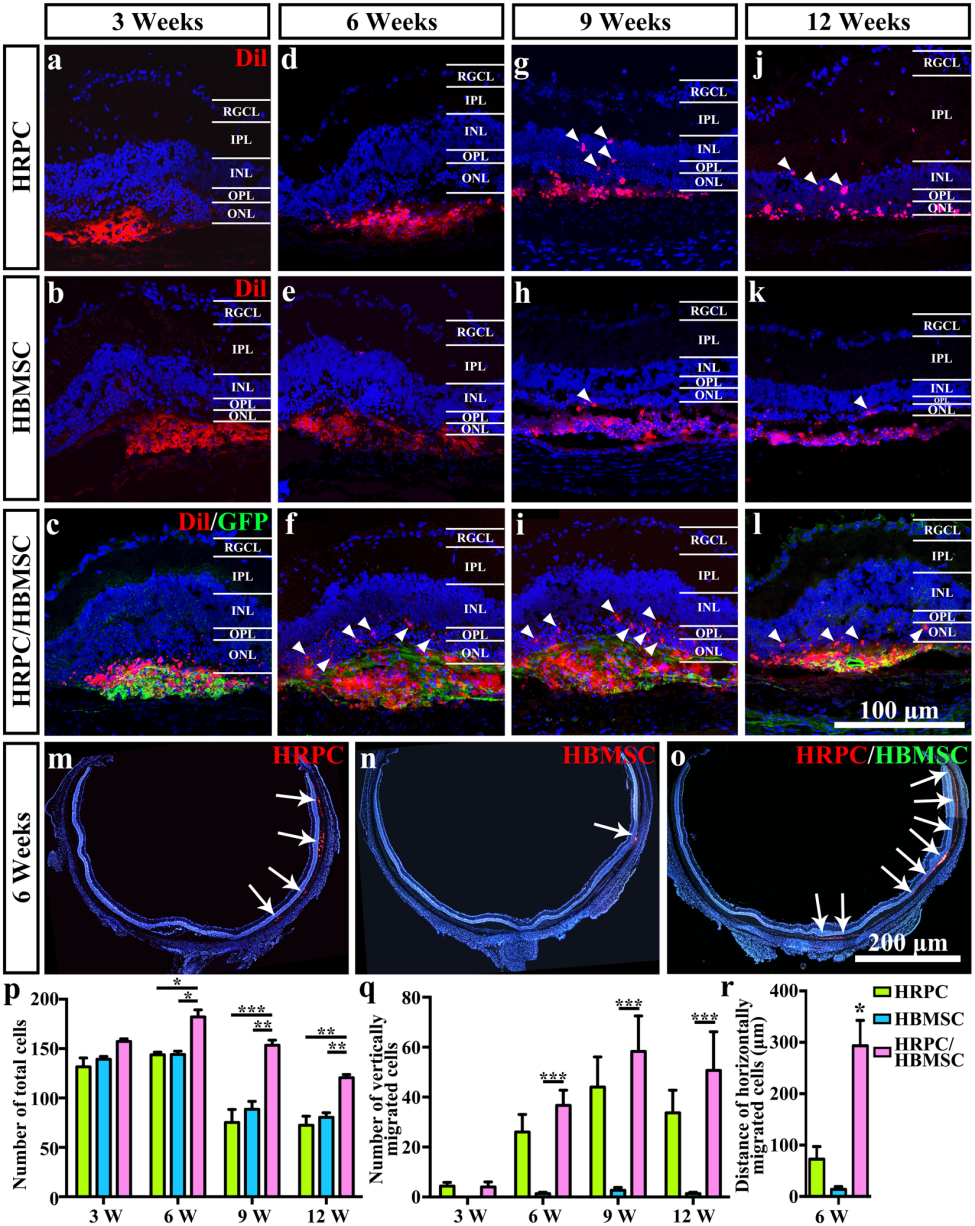
Comparison of cellular migration after single and combined cell transplantation into subretinal space (SRS). (**a**–**l**) Vertical cellular migration of human retinal progenitor cells (HRPCs) and (or) human bone mesenchymal stem cells (HBMSCs) after single and combined cell transplantation to SRS on 3, 6, 9 and 12 post operational weeks. Results showed that vertical cellular migration started earlier in combined transplantation group. Also, more vertically migrated cells could be found in combined transplantation group. (**m**–**o**) Horizontal cellular migration of HRPCs and HBMSCs after single and combined cell transplantation to SRS on 6 post operational weeks. Results showed that more and broader horizontal cellular migration could be visualized in combined transplantation group. (**p**–**q**) Corresponding statistical analysis of (**a**–**l)**. (**r**) Corresponding statistical analysis of (**m**,**n)**. *P < 0.05; **P < 0.01; ***P < 0.001. (n = 3).

After HRPCs, HMSCs and their combination were individually transplanted to the SRS of RCS rats, and cells were counted among three groups. Our results showed that combined transplantation presented a significant increase in the number of cells by 6, 9 and 12 weeks post-operation when compared with single transplantation groups (Fig. 5p). This result indicates that cells survived better under our combined transplantation regimen. To assess the change more specifically, the numbers of transmitted cells were measured both longitudinally and latitudinally. During the 3-week to 12-week post-transplantation period, cells gradually migrated from the SRS into the inner retina (Fig. 5a–l). At 3 weeks post-operation, there was no obvious longitudinal migrations in all 3 groups (Fig. 5a–c). The combined transplantation group started to show significant migration by 6 weeks post-operation (Fig. 5f,q). However, the HRPCs and HBMSCs single transplantation groups did not display migration until 9 weeks post-operation (Fig. 5d,e,g,h). At that time, the combined transplantation group showed a significant increase of migrated cells in the inner retina (Fig. 5i,q). By 12 weeks, although the number of migrated cells in the combined transplantation group decreased, it still presented significantly high trends compared to the single transplantation groups (Fig. 5j–l,q). We also selected 6 weeks post-transplantation to measure the latitudinal migration of transplanted cells. Our results showed that rare migration occurred in the HBMSCs transplantation group (Fig. 5n,r). Although cells migrated in the HRPCs transplantation group, the number of migrated cells was significantly lower compared to that in the combined transplantation group (Fig. 5m,o,r). Taken together, our results demonstrated that cells in the combined transplantation group migrated better than in the single transplantation groups.

### Photoreceptor differentiation of transplanted cells

**Figure 6:**
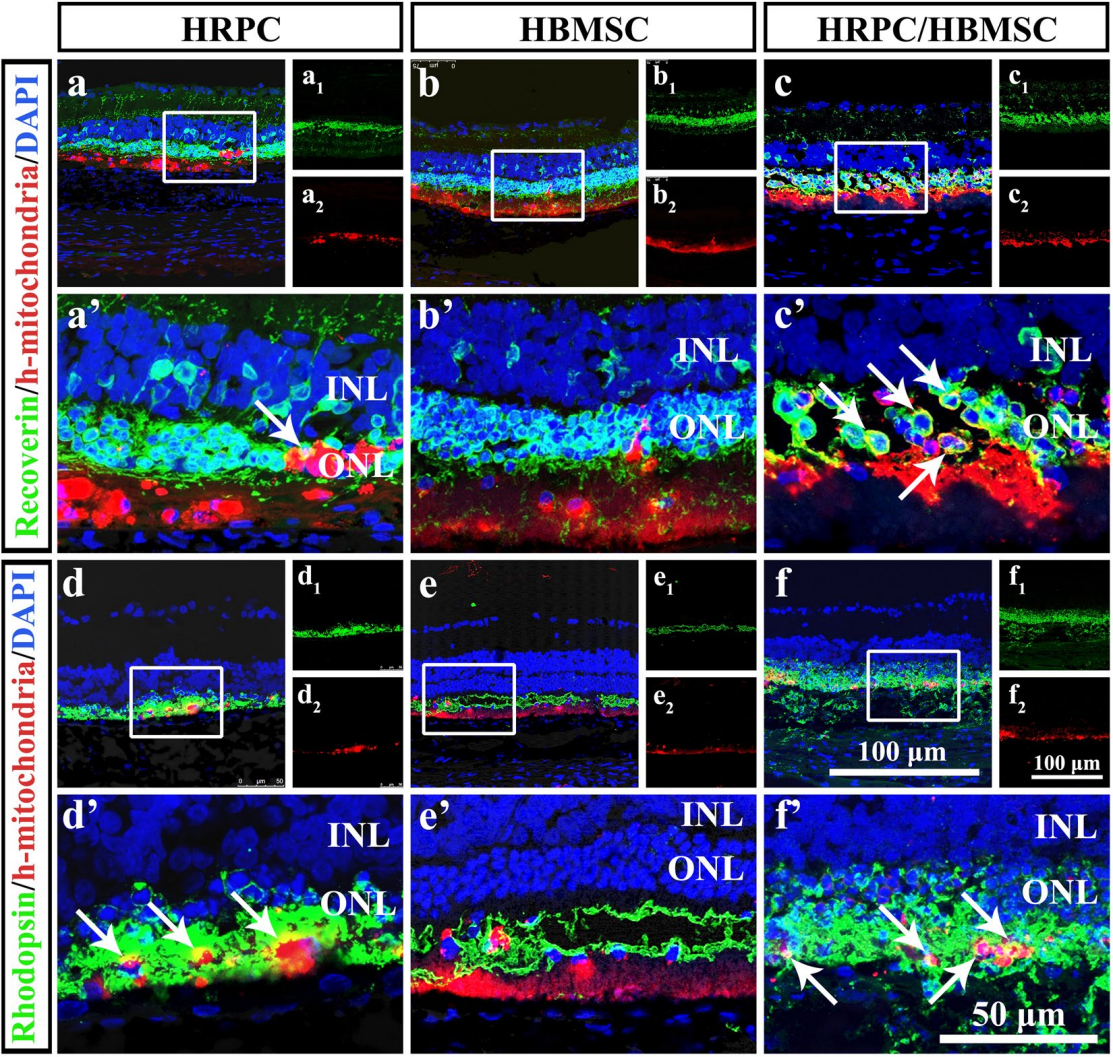
Cellular differentiation after single and combined cell transplantation into subretinal space (SRS) on 6 post operational weeks. (**a**–**c**) Confocal analysis of Recoerin and human mitochondria double staining after single and combined transplantation. (a_1_,b_1_,a_2_,b_2_) Corresponding Recoerin and human mitochondria single staining. (a’–c’) Enlarged area reflecting the differentiation of transplanted cells. (**d**–**f**) Confocal analysis of Rhodopsin and human mitochondria double staining after single and combined transplantation. (d_1_–f_1_,d_2_–f_2_) Corresponding Rhodopsin and human mitochondria single staining. (d’–f’) Enlarged area reflecting the differentiation of transplanted cells. Results showed that there are more differentiation happened in HRPCs and HBMSCs combined transplantation group. (n = 3).

Since the total number of transplanted cells reached the highest level by 6 weeks post-operation (Fig. 5p), the differentiation of transplanted cells was measured at this time point. Using photoreceptor marker recoverin, we found that in the HRPC single transplantation group, there was co-expression of recoverin and h-mitochondria, indicating that the transplanted cells differentiated into photoreceptors (Fig. 6a,a’). However, this differentiation was seldom observed (Fig. 6a,a’). In the HBMSC single transplantation group, there was no co-expression of recoverin and h-mitochondria observed (Fig. 6b,b’). In contrast, there were many more cells co-expressing recoverin and h-mitochondria in the combined transplantation group compared to those in the HRPC single transplantation group, indicating that transplanted cells differentiated into photoreceptors more often under our combined transplantation regimen (Fig. 6c,c’). To confirm the results, we also used rhodopsin. Our results were consistent with the recoverin staining assays (Fig. 6d–f,d’–f’). The differences were that more cells co-expressed rhodopsin and h-mitochondria under HRPC single and combined transplantations, indicating that more differentiation occurred.

### Inflammatory modulation of transplanted cells

**Figure 7:**
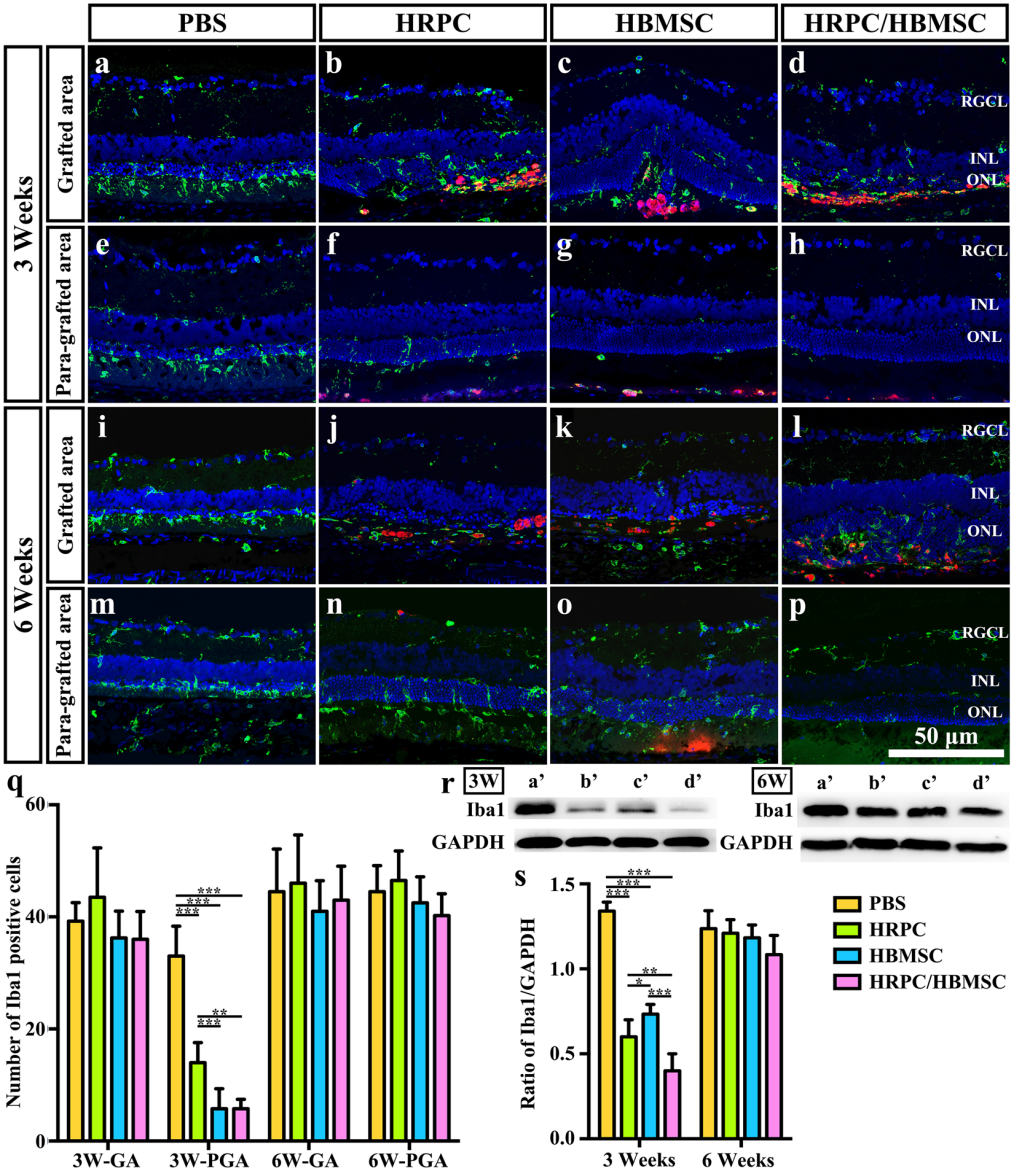
Comparison of microglia activation after single and combined cell transplantation into subretinal space (SRS). (**a**–**d**) Representative image of Iba1-staining microglia within grafted area after PBS, human retinal progenitor cells (HRPCs), human bone mesenchymal stem cells (HBMSCs) and combined cells injection on 3 post operational weeks. (**e**–**h**) Representative image of Iba1-staining microglia within para-grafted area after PBS, HRPCs, HBMSCs and combined cells injection on 6 post operational weeks. (**i**–**l**) Representative image of Iba1-staining microglia within grafted area after PBS, HRPCs, HBMSCs and combined cells injection on 3 post operational weeks. (**m**–**p**) Representative image of Iba1-staining microglia within para-grafted area after PBS, HRPCs, HBMSCs and combined cells injection on 6 post operational weeks. (**q**) Corresponding statistical analysis of (**a**–**p)**. GA: grafted area. PGA: Para-grafted area. (**r**) Western blot analysis of Iba1 protein on post operational 3 and 6 weeks (a’: PBS; b’: HRPCs; c’: HBMSCs; d’: HRPCs/HBMSCs). For original uncropped images, please see Supplementary Figs 2 and 3. (**s**) Corresponding statistical analysis of (**r**). *P < 0.05; **P < 0.01; ***P < 0.001. (n = 3).

Immunoreactions and inflammation can influence retinal function in many ways, and microglia cells play an important role^39, 40^. We evaluated the number of Iba1-immunoreactive retinal microglia cells in the PBS injection, HRPC transplantation, HBMSC transplantation and HPRC/HBMSC combined transplantation groups. Our results showed that there were no significant differences among these four groups in the cell-grafted areas by 3 post operational weeks (Fig. 7a–d). However, there were significant differences in the para-grafted areas at this time point (Fig. 7e–h). Cell-transplanted groups all displayed significant decreases in microglia cell number. More importantly, the HBMSC transplantation group and the combined transplantation groups showed more effective inflammation suppression compared with the HRPC transplantation group (Fig. 7f–h,q). These differences were not found to be statistically significant, however (Fig. 7g,h,q). To verify this finding, a western blot analysis of Iba1 expression was performed. By 3 post operational weeks, our results showed that the Iba1 expression level was significantly decreased in all three groups (Fig. 7r,s). The combined transplantation groups showed significant decreased Iba1 expression levels compared to the HRPC and HBMSC transplantation group (P = 0.016298 and P = 0.000988). The HRPC transplantation group showed decreased Iba1 expression levels compared to the HBMSC transplantation group (Fig. 7r,s). By 6 post operational weeks, no significant differences could be detected among all groups in either the grafted or para-grafted areas (Fig. 7i–p). Western-blot analysis results were consistent with this finding (Fig. 7r,s). In addition, we also assayed the expression levels of inflammatory factors and neurotrophic factors with RT-PCR. Our results showed that all 3 types of cell transplantation decreased TNF and IL-1β expression levels and increased NGF and BDNF expression levels (Supplementary Fig. 1a–d). These differences were strongest in the cell transplantation groups containing HBMSCs (Supplementary Fig. 2a–d).

### The combined cell transplantation suppressed Müller cells

**Figure 8:**
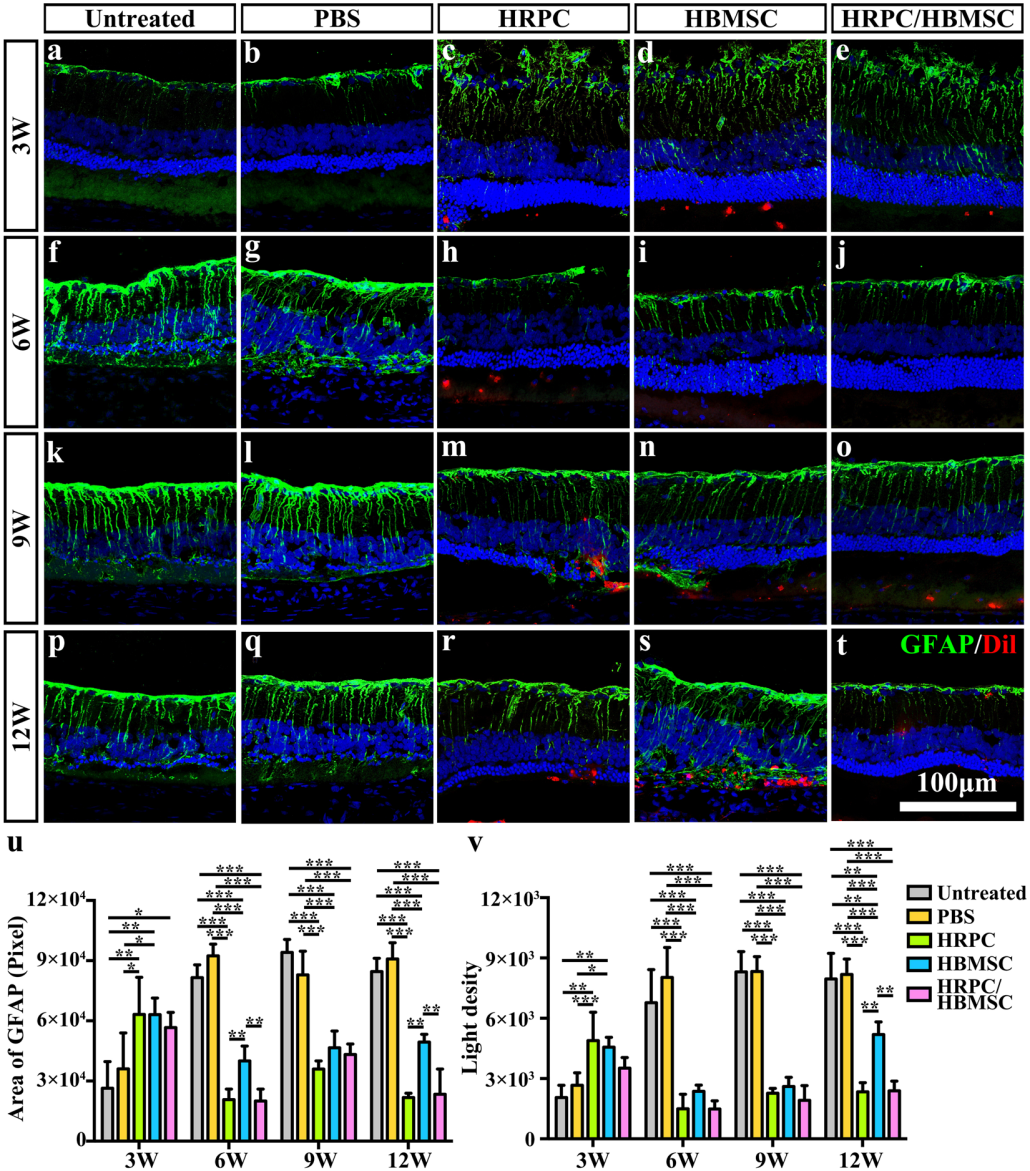
Comparison of retinal gliosis after single and combined cell transplantation into subretinal space (SRS). (**a**–**t**) Representative image of GFAP-staining Müller cells in untreated group and within grafted area after PBS, human retinal progenitor cells (HRPCs), human bone mesenchymal stem cells (HBMSCs) and combined cells injection on corresponding 3, 6, 9 and 12 post operational weeks. (**u**) Statistical analysis of the area of total GFAP positive cells the in all 5 groups at 4 time points. (**v**) Statistical analysis of the light density of GFAP positive cells the in all 5 groups at 4 time points. *P < 0.05; **P < 0.01; ***P < 0.001. (n = 3).

To evaluate the gliosis state after cell transplantation, GFAP counter-staining was performed in the untreated, PBS injection, HRPC transplantation, HBMSC transplantation and HPRC/HBMSC combined transplantation groups. There showed no significant difference between untreated group and PBS injection group on all time points (Fig. 8a,b,f,g,k,l,p,q,u,v). After 3 weeks, the cell-transplantation groups presented a more severe gliosis state than PBS injection group and untreated group (Fig. 8a–e,u,v). However, the gliosis state is significantly overturned in all three transplantation groups by 6 weeks post-operation. The gliosis was obviously reduced in all cell-transplantation groups but increased in the PBS injection group and untreated group (Fig. 8f–j,u,v). After 9 weeks, gliosis remained at a high level in the PBS injection group and untreated group and increased moderately in the cell-transplantation groups (Fig. 8k–o,u,v). As time went on, gliosis in the HBMSC transplantation group increased significantly after 12 weeks, while gliosis in the HRPC and combined transplantation groups remained in a low level (Fig. 8p–t,u,v). The western blot results also confirmed the findings (Supplementary Fig. 3).

## Discussion

Retinal degenerative diseases, including RP and age-related macular degeneration, are far from being fully understood and treated. Promising treatments include those focused on gene, drug and stem cell therapies^41^. There are many different types of stem cells that can be selected as candidate cells^42^. In addition, stem cell transplantation can perform a variety of functions including cell replacement, anti-hazardous substances, trophic action, neural plasticity and immune modulation^43^. Taken together, transplantation of stem cells possesses huge potential in treating degenerative disease. However, the characteristics of one particular stem cell type is fixed, which means that the effects of transplantation of a single cell type is limited. In this way, to integrate the different functions of stem cell transplantation, the combined transplantation of two or more types of cells can be implemented^32–37, 44^. Ranging from mice and rats to cats and chickens, many animal models have been used to mimic the process of RP. However, the RCS rat is the classic naturally occurring inherited retinal degenerative disease model^45^. In the present study, HRPCs and HBMSCs were combined and transplanted into the SRS of RCS rats to show the effects of combined transplantation.

ERG tests showed that the visual function in all groups declined over time. Furthermore, combined transplantation did not present an advantage over single transplantation until 6 weeks post-operation. This effect lasted at least through 12 weeks post-transplantation. Single transplantation of HRPCs and HBMSCs showed no significant differences between each other, illustrating the same rescue effects of these two cells. By 12 weeks post-operation, neither HRPCs nor HBMSCs alone preserved visual function. In other HRPCs single transplantation experiments, ERG testing showed better results at 12 weeks^24^. This might be due to different states and amounts of HRPCs used for transplantation. However, though HRPCs and HBMSCs lost their rescue effect in our experiments, the combined transplantation of both cell types maintained visual function better, indicating the mutual promotion effect of these two cell types. Interestingly, though without achieving statistical significance, HBMSCs single transplantation showed a high to low rescue effect compared with HRPCs single transplantation. This result indicates the different mechanisms in preserving visual function between HRPCs and HBMSCs.

In retina transplantation research, the thickness of ONL is commonly used to measure the degree of photoreceptor protection^24, 46, 47^. Our study showed that the HBMSCs and combined transplantation groups displayed thicker ONL at 3 weeks. However, it decreased more quickly in the HBMSCs group with time. In later time points, 9 and 12 weeks post-operation, the HRPCs group showed better photoreceptor protection than the HBMSCs group did. However, the combined transplantation group still displayed the best protection effect, especially in the late stage of transplantation. This may be due to the complementary effect of HBMSCs and HRPCs, where the former cells create better microenvironments and the latter cells play a major role in repairing the retina.

To best perform their cell replacement function, transplanted cells should migrate into the inner retina. However, in a previous HRPCs SRS transplantation study, a low migration rate was observed^48^. In the present study, increases of both longitudinal and latitudinal migration were discovered under a combined transplantation regimen. Chemokines played a significant role in helping migration of the cells. As the major role of HBMSCs in rescuing retinal damage is to secret factors and improve the microenvironment^29–31^, combined transplantation may actually coordinate the characteristics of these two cells and further facilitate HRPCs migration.

Differentiation of transplanted cells is the most important but difficult sign of cell replacement. In the photoreceptor degeneration model, differentiation of transplanted cells into photoreceptors is an exciting achievement. In single transplantation groups, HRPCs were able to differentiate into recoverin- and rhodopsin-positive photoreceptors while HBMSCs could not, illustrating the different functions of these two cells. These results are similar to what has been shown in a previous study^49^. In our combined transplantation tests, the differentiation of HRPCs increased compared to single transplantation, indicating that HBMSCs helped HRPCs to differentiate. As acquisition of HBMSCs was through a well-established clinical procedure^49^, the combined transplantation of HBMSCs and HRPCs is realistic for clinical application.

Our previous studies showed that activation of microglia may lead to a vicious cycle^39, 40^ and that SRS transplantation of neural stem cell was able to suppress this activation^20^. In the present study, we found that all SRS transplantations actually suppressed the microglial activation. This effect is only restricted to the para-grafted area by post-operational week 3. Additionally, we showed no significant difference between HBMSCs single transplantation and combined transplantation. Unfortunately, the immune suppressing effect of HRPCs is less than that of HBMSCs. Taken together, HBMSCs played a major role in the immune regulation of SRS transplantation^50^, which might support the vitality of HRPCs. The limited immune suppressing effect might be due to the limited number of transplanted cells or might be an irreversible effect of severe photoreceptor degeneration.

Gliosis is the adaptive response of Müller cells that may further impede the differentiation and integration of transplanted cells^51^. Our study found transplantation of HRPCs and HBMSCs singly or combined was able to decrease the gliosis levels at early stages, approximately 3–9 weeks post-transplantation. However, HBMSCs single transplantation resulted in a reduced gliosis-suppressing effect at 12 weeks post-operation, while HRPCs single and combined transplantation still inhibited gliosis well at this time point, indicating that HRPCs are better than HBMSCs at performing the gliosis-suppressing effect. A previously published study found that the transplantation of HBMSCs into the retina caused gliosis^52^, which could be the reason why GFAP levels increased again in the later stage of HBMSCs transplantation. More importantly, combined transplantation still maintained a gliosis-suppressing state, providing more strong evidence for the benefits of mutual coordination in our combined transplantation protocol.

## Materials and Methods

### Ethical approval on human tissue

All the experimental procedures throughout the present study involving human tissue were approved by the Human and Animal Research Ethics Committees of the Third Military Medical University. The research adhered to the tenets of the Declaration of Helsinki and written informed consent and surgical consent were obtained from all patients (WHO Trial Registration, ChiCTR-TNRC-08000193). Participants provided their written consent and approved the consent procedure for our studies.

### Animal and ethics

The RCS rats were kindly donated by the Becjnab Vision Center and were raised in a specific-pathogen-free room and maintained on a 12-h light/dark cycle. Rats were supplied by the Animal Care Centre of Southwest Hospital. All tissue collection and experimental procedures were performed according to protocols approved by the Institutional Review Board of the Third Military Medical University and conformed to the National Institutes of Health (NIH) guidelines on the ethical use of animals (for more information, please see supplementary ethical statement).

### Isolation and culture of HRPCs

HRPCs were harvested from human fetal eyes between 11–13 weeks of gestation. The neuroretinas were separated as previously described^1, 53^. Fetal neuroretinas were cut into pieces, rinsed, and digested for 20–30 min with 1 U/ml papain at 37 °C. After a 10-second agitation, the cell suspension was centrifuged at 1000 rpm for 5 min. Any remaining undissociated tissue was processed again. Then, cell pellets were re-suspended in culture medium containing 10 ng/mL epidermal growth factor, 20 ng/mL bFGF, 2 mM L-glutamine, 1% Penicillin/Streptomycin (P/S; Invitrogen), and 5% fetal bovine serum (FBS, Invitrogen). The number of live and dead cells was counted using a trypan blue assay (Sigma-Aldrich). The isolated cells were plated onto fibronectin-coated (100 μg/mL) tissue culture flasks at a density of 9–13 × 10^3^ cells/cm^2^ and were incubated in a 5% CO_2_ saturation-humidity atmosphere in 37 °C. Twenty-four hours after plating, FBS was removed by changing the culture medium. The viability of purified cells was determined using trypan blue staining and only cell preparations with greater than 95% viability in passage 2 were used for transplantation.

### Isolation and purification of HMSCs

HMSCs were isolated from the bone marrow aspirates of donors (20–30 years old). The marrow was diluted with phosphate buffered saline (PBS) (pH 7.3) and loaded over a Ficoll solution with a density of 1.073 g/ml. After a 20 min centrifugation at 900 g, nucleated cells were collected and washed with PBS at room temperature^54^. The precipitate was re-suspended in MSC culture medium, containing DMEM/F12, 10% FBS (Hyclone). Cells were cultured up to 5 passages. At passage 3, the identification of HMSCs was tested by FACS.

### Cell transplantation to RP animal model

Sub-retinal transplantation was performed as previously described^55^. In brief, HRPCs, HBMSCs or their combination were injected into the SRS of the left eyes of RCS rats at the age of postnatal 3 weeks (12/group). The identical volume of 0.01 M PBS was injected into the SRS of the right eyes of RCS rats (12/group). In the HRPCs and HBMSCs singly transplanted groups, all transplanted cells were labeled with the fluorescent marker CM-DiI (2 mg/ml, Invitrogen). Cell suspensions (5 μl/eye, total 4 × 10^5^ cells/eye) were slowly injected into the left temporal SRS. Then, 5 μl 0.01 M PBS was slowly injected into the right temporal SRS. In the HRPCs and HBMSCs combined transplanted group, HRPCs were labeled with CM-DiI and HBMSCs were labeled with GFP via lentivirus infection. Cell suspension mixtures (5 μl/eye, total 4 × 10^5^ cells/eye) containing HRPCs (2 × 10^5^ cells/eye) and HBMSCs (2 × 10^5^ cells/eye) were slowly injected into the left temporal SRS. Then, 5 μl 0.01 M PBS was slowly injected into the right temporal SRS. The pupils were dilated with 1% of tropicamide (Santen Pharmaceutical Co., Ltd. Osaka, Japan) 30 mins before the surgery. A 10 μl Hamilton syringe (30 gauge; Hamilton, Nevada, USA) containing the cell suspension was tangentially inserted into the SRS through the conjunctiva and sclera, causing a self-sealing wound tunnel. The edge of cornea was punctured to reduce intraocular pressure and limit the efflux of cells at the injection site. Fundus examinations were performed via direct ophthalmoscope viewing after the operations.

### ERG Recording

ERG was performed on 3, 6, 9 and 12 post-operation weeks to evaluate the retinal function changes as we previously described. In brief, RCS rats were dark adapted for at least 12 hours prior to conduct ERG^56^. Anesthetization were performed by intraperitoneal injection a solution of ketamine (120 mg/kg) and xylazine (20 mg/kg). Pupils were dilated using 1% of tropicamide. Body temperature of animals was maintained at 37 °C by a bottom heating pad to prevent hypothermia. Two active gold electrodes were placed on each cornea as the recording electrodes. The reference and ground electrodes were placed subcutaneously in the mid-frontal area of head and the tail, respectively. Light stimulations were delivered with a xenon lamp at 3.0 cd·s/m^2^. The amplitudes of a- and b-waves were recorded and processed by the RETI-Port device (Roland consult, Brandenburg, Germany). All the procedures were performed in a dark room with dim red safety light.

### Tissue preparation and Immunofluorescence

After being anesthetized by 1% pentobarbital (150 mL/kg), RCS rats were perfused with normal saline and 4% paraformaldehyde via the circulation system on 3, 6, 9 and 12 post-operation weeks as we previously described^40^. Eyeballs were enucleated and fixed in 4% paraformaldehyde for 3 hours. After incubation in 30% glucose solution overnight, retinal tissues were collected and serially frozen-sectioned to a thickness of 10 μm. Immunofluorescence was performed as previously described^57^. In detail, sections that crossed the optic disc were rinsed in 0.1 M PBS and blocked for 30 min in 10% of goat serum diluted in 0.1% of Triton X-100. Then, sections were incubated with the primary antibodies, anti-human mitochondrial antibody (1:200, mouse, Abcam), anti-human mitochondrial antibody (1:200, rabbit, Millipore, Billerica, MA), anti-recoverin (1:1000, rabbit, Millipore), anti-rhodopsin (1:8000; rabbit, Sigma-Aldrich), anti-Iba1 (1:500; Wako, Japan) and GFAP (1:500, rabbit, Sigma Chemical Co) in 1% BSA at 4 °C overnight. Secondary antibodies, cy3-or 488-conjugated (Invitrogen), were then implemented (1:400, 37 °C, 2 h). Some sections were processed only with the secondary antibodies as negative controls. Before examination with a confocal laser scanning microscope (Leica, Germany), sections were counterstained with DAPI (Sigma Aldrich, St. Louis, MO, USA).

### Quantitative RT-PCR

The retinas of RCS and rdy rats were collected at postnatal week 3 to evaluate mRNA levels using quantitative RT-PCR (qRT-PCR). Total mRNA was extracted using the RNeasy Mini kit (Qiagen, ML, USA) and cDNA synthesis was conducted using SuperScript III First-Strand Synthesis SuperMix (Invitrogen, CA, USA). The procedures of mRNA extraction and cDNA synthesis were performed according to manufacturer’s instructions. Primers are listed in Supplementary Table. Reactions were performed in a 25 μl Eppendorf tube with 2 μl of cDNA, 0.3 μl of forward and reverse primer (10 pmol/μl), 10 μl of 2× Mix (full velocity SYBR green qPCR master mix, Stratagene) Taq and 7.4 μl of ddH_2_O. The procedure for real-time qRT-PCR included 4 min at 94 °C, followed by 30 cycles of 30 s at 94 °C, 30 s at 60 °C, and 30 s at 72 °C (Roche LC480, Roche Applied Science). All of the qRT-PCR reactions were performed in triplicate for yielding averaged Ct values. Expression (evaluated as fold change for each target gene) was normalized to GAPDH in microglial cells following the well-established Δ-Δ method^58, 59^. Data were presented as fold change over the control. A non-template control was included in the experiment to estimate DNA contamination of isolated mRNA and reagents.

### Western blot

Animals were euthanized with CO2 at postoperative weeks 6 and 12. Eyeballs were enucleated and retinas were quickly isolated on ice. After being rinsed in 0.01 M PBS and drained, the retinas were lysed in ice-cold tissue lysis buffer (10% PMSF + 90% RIPA). The lysates were then cleared by centrifugation at 15,000 g for 6 min at 4 °C. Protein concentration was determined using the BCA Protein Assay (Beyotime, China) according to the manufacturer’s instructions. Total proteins (10 μg per slot) were electrophoresed on a 12% sodium dodecyl sulfate polyacrylamide gel and then transferred onto polyvinylidene fluoride membranes. After blocking in 5% fat-free milk for 2 h at 37 °C, membranes were incubated with anti-Iba1 (1:500; Wako, Japan), anti-GFAP antibody (1:500, rabbit, Sigma Chemical Co) and anti-GADPH (1:1000, mouse, Proteintech Group, Chicago) antibody overnight at 4 °C. The next day, membranes were further incubated with peroxidase-conjugated immuno-globulin G (1:2000; Santa Cruz Biotechnology). Finally, membranes were scanned using the Bio-rad exploding system (Bio-rad, CA, USA) with corresponding software.

### ONL thickness analysis

Three DAPI-stained sections in each group that were cut using the same horizontal angle across the optic disc were chosen to measure the thickness of the ONL. In each section, areas in the middle of the transplanting site and the optic disc were selected. The thickness of the ONL was measured by Image J (NIH, USA).

### Cell counting and light density analysis

To quantitatively analyze the changes in marker expression of HRPCs during passaging, 3 comparable visual fields in each cell slide were randomly selected. There were 3 repeats in each passage. The numbers of Ki67, PAX6, SOX2, Nestin and GFAP positive cells were manually counted and averaged. To quantitatively analyze the cell migration after cell transplantation, at least 3 sections across the optic disc in each rat were selected. Under the same magnification, Dil and GFP positive vertically migrated and total cells at transplanted sites were manually counted and averaged. Simultaneously, Dil and GFP positive horizontally migrated cells on the whole retina sections were manually counted and averaged. To quantitatively analyze the changes of microglia cells, at least 3 sections across the optic disc in each rat were selected. Both the visual fields in the transplanted area and para-transplanted area (areas in the middle of the transplanted site and the optic disc) were selected. The number of Iba1-positive cells were manually counted and averaged for each visual field. Light density of GFAP-positive cells was recorded by ImageJ (NIH).

### Statistical analysis

Using the Statistical Product and Service Solutions software V17.0 (SPSS, Chicago, IL, USA), all quantitative results were analyzed by one-way ANOVA analyses of variance and Whitney-Mann U test. The data were presented as the mean ± standard error. P < 0.05 was considered statistically significant.

## Electronic supplementary material

Supplementary Information

## References

[1] Hartong DT, Berson EL, Dryja TP (2006). Retinitis pigmentosa. Lancet.

[2] Bunker CH, Berson EL, Bromley WC, Hayes RP, Roderick TH (1984). Prevalence of retinitis pigmentosa in Maine. Am. J. Ophthalmol..

[3] Milam AH, Li ZY, Fariss RN (1998). Histopathology of the human retina in retinitis pigmentosa. Prog. Retin. Eye Res..

[4] Berson EL (1993). A randomized trial of vitamin A and vitamin E supplementation for retinitis pigmentosa. Arch. Ophthalmol..

[5] Berson EL (2004). Further evaluation of docosahexaenoic acid in patients with retinitis pigmentosa receiving vitamin A treatment: subgroup analyses. Arch. Ophthalmol..

[6] Kumar A (2014). Efficacy of oral valproic acid in patients with retinitis pigmentosa. J. Ocul. Pharmacol. Ther..

[7] Weissmiller AM, Wu C (2012). Current advances in using neurotrophic factors to treat neurodegenerative disorders. Transl. Neurodegener..

[8] Viringipurampeer IA, Bashar AE, Gregory-Evans CY, Moritz OL, Gregory-Evans K (2013). Targeting inflammation in emerging therapies for genetic retinal disease. Int. J. Inflam..

[9] Guadagni V, Novelli E, Piano I, Gargini C, Strettoi E (2015). Pharmacological approaches to retinitis pigmentosa: A laboratory perspective. Prog. Retin. Eye Res..

[10] Gonzalez-Cordero A (2013). Photoreceptor precursors derived from three-dimensional embryonic stem cell cultures integrate and mature within adult degenerate retina. Nat. Biotechnol..

[11] MacLaren RE (2006). Retinal repair by transplantation of photoreceptor precursors. Nature.

[12] Pearson RA (2012). Restoration of vision after transplantation of photoreceptors. Nature.

[13] Shirai H (2016). Transplantation of human embryonic stem cell-derived retinal tissue in two primate models of retinal degeneration. Proc. Natl. Acad. Sci. USA.

[14] Lamba DA, Gust J, Reh TA (2009). Transplantation of human embryonic stem cell-derived photoreceptors restores some visual function in Crx-deficient mice. Cell Stem Cell.

[15] Perez VL, Saeed AM, Tan Y, Urbieta M, Cruz-Guilloty F (2013). The eye: A window to the soul of the immune system. J. Autoimmun..

[16] Dahlmann-Noor A, Vijay S, Jayaram H, Limb A, Khaw PT (2010). Current approaches and future prospects for stem cell rescue and regeneration of the retina and optic nerve. Ca. J. Ophthalmol..

[17] Huo SJ (2012). Transplanted olfactory ensheathing cells reduce retinal degeneration in Royal College of Surgeons rats. Curr. Eye Res..

[18] Jian Q, Li Y, Yin ZQ (2015). Rat BMSCs initiate retinal endogenous repair through NGF/TrkA signaling. Exp. Eye Res..

[19] Li F, Zeng Y, Xu H, Yin ZQ (2015). Subretinal transplantation of retinal pigment epithelium overexpressing fibulin-5 inhibits laser-induced choroidal neovascularization in rats. Exp. Eye Res..

[20] Li Z (2016). Neural stem cells transplanted to the subretinal space of rd1 mice delay retinal degeneration by suppressing microglia activation. Cytotherapy.

[21] Qiu G (2005). Photoreceptor differentiation and integration of retinal progenitor cells transplanted into transgenic rats. Exp. Eye Res..

[22] Tao, Z. *et al.* Lin28B promotes muller glial cell de-differentiation and proliferation in the regenerative rat retinas. *Oncotarget*, doi:10.18632/oncotarget.10343 (2016). 10.18632/oncotarget.10343 PMC5226514 27384999

[23] Aftab U (2009). Growth kinetics and transplantation of human retinal progenitor cells. Exp. Eye Res..

[24] Luo J (2014). Human retinal progenitor cell transplantation preserves vision. J. Biol. Chem..

[25] Tzameret A (2014). Transplantation of human bone marrow mesenchymal stem cells as a thin subretinal layer ameliorates retinal degeneration in a rat model of retinal dystrophy. Exp. Eye Res..

[26] Tzameret A (2015). Epiretinal transplantation of human bone marrow mesenchymal stem cells rescues retinal and vision function in a rat model of retinal degeneration. Stem Cell Res..

[27] Inoue Y (2007). Subretinal transplantation of bone marrow mesenchymal stem cells delays retinal degeneration in the RCS rat model of retinal degeneration. Exp. Eye Res..

[28] Arnhold S, Absenger Y, Klein H, Addicks K, Schraermeyer U (2007). Transplantation of bone marrow-derived mesenchymal stem cells rescue photoreceptor cells in the dystrophic retina of the rhodopsin knockout mouse. Graefes Arch. Clin. Exp. Ophthalmol..

[29] Kicic A (2003). Differentiation of marrow stromal cells into photoreceptors in the rat eye. J. Neurosci..

[30] Oh JY (2008). The anti-inflammatory and anti-angiogenic role of mesenchymal stem cells in corneal wound healing following chemical injury. Stem Cells.

[31] Baglio SR, Pegtel DM, Baldini N (2012). Mesenchymal stem cell secreted vesicles provide novel opportunities in (stem) cell-free therapy. Front. Physiol..

[32] Yamahara K (2008). Augmentation of neovascularization [corrected] in hindlimb ischemia by combined transplantation of human embryonic stem cells-derived endothelial and mural cells. PLoS One.

[33] Park DY (2013). Combined Transplantation of Human Neuronal and Mesenchymal Stem Cells following Spinal Cord Injury. Global Spine J..

[34] Ao Q (2007). Combined transplantation of neural stem cells and olfactory ensheathing cells for the repair of spinal cord injuries. Med. Hypotheses.

[35] Ohmura Y (2010). Combined transplantation of pancreatic islets and adipose tissue-derived stem cells enhances the survival and insulin function of islet grafts in diabetic mice. Transplantation.

[36] Souza, L. C. *et al.* Combined transplantation of skeletal myoblasts and mesenchymal cells (cocultivation) in ventricular dysfunction after myocardial infarction. *Arq. Bras. Cardiol.* **83**, 294–299, 288–293 (2004). 10.1590/s0066-782x2004001600004 15517043

[37] Lv YT (2013). Transplantation of human cord blood mononuclear cells and umbilical cord-derived mesenchymal stem cells in autism. J. Transl. Med..

[38] Pittenger MF (1999). Multilineage potential of adult human mesenchymal stem cells. Science.

[39] Jin, N., Gao, L., Fan, X. & Xu, H. Friend or Foe? Resident Microglia vs Bone Marrow-Derived Microglia and Their Roles in the Retinal Degeneration. *Mol. Neurobiol.*, doi:10.1007/s12035-016-9960-9 (2016). 10.1007/s12035-016-9960-9 27318678

[40] Gao L (2015). Neuroprotective effect of memantine on the retinal ganglion cells of APPswe/PS1DeltaE9 mice and its immunomodulatory mechanisms. Exp. Eye Res..

[41] Veleri S (2015). Biology and therapy of inherited retinal degenerative disease: insights from mouse models. Dis. Model. Mech.

[42] Jin ZB, Okamoto S, Mandai M, Takahashi M (2009). Induced pluripotent stem cells for retinal degenerative diseases: a new perspective on the challenges. J. Genet..

[43] Fan X (2014). Stem-cell challenges in the treatment of Alzheimer’s disease: a long way from bench to bedside. Med. Res. Rev..

[44] Tang ZP (2010). Combined transplantation of neural stem cells and olfactory ensheathing cells improves the motor function of rats with intracerebral hemorrhage. Biomed. Environ. Sci..

[45] Rivas MA, Vecino E (2009). Animal models and different therapies for treatment of retinitis pigmentosa. Histol. Histopathol..

[46] Tian C, Weng CC, Yin ZQ (2010). BDNF improves the efficacy ERG amplitude maintenance by transplantation of retinal stem cells in RCS rats. Adv. Exp. Med. Biol..

[47] Abe T (2005). Protection of photoreceptor cells from phototoxicity by transplanted retinal pigment epithelial cells expressing different neurotrophic factors. Cell Transplant.

[48] Unachukwu UJ (2016). Predicted molecular signaling guiding photoreceptor cell migration following transplantation into damaged retina. Sci. Rep..

[49] Tomita M (2006). A comparison of neural differentiation and retinal transplantation with bone marrow-derived cells and retinal progenitor cells. Stem Cells.

[50] Xian B, Huang B (2015). The immune response of stem cells in subretinal transplantation. Stem Cell Res. Ther..

[51] Hippert C, Graca AB, Pearson RA (2016). Gliosis Can Impede Integration Following Photoreceptor Transplantation into the Diseased Retina. Adv. Exp. Med. Biol..

[52] Tassoni A, Gutteridge A, Barber AC, Osborne A, Martin KR (2015). Molecular Mechanisms Mediating Retinal Reactive Gliosis Following Bone Marrow Mesenchymal Stem Cell Transplantation. Stem Cells.

[53] Strettoi E (2015). A Survey of Retinal Remodeling. Front. Cell. Neurosci..

[54] Ramsden CM (2013). Stem cells in retinal regeneration: past, present and future. Development.

[55] Shintani K, Shechtman DL, Gurwood AS (2009). Review and update: current treatment trends for patients with retinitis pigmentosa. Optometry.

[56] Wang R, Jiang C, Ma J, Young MJ (2012). Monitoring morphological changes in the retina of rhodopsin−/− mice with spectral domain optical coherence tomography. Invest. Ophthalmol. Vis. Sci..

[57] Gao L (2016). Intermittent high oxygen influences the formation of neural retinal tissue from human embryonic stem cells. Sci. Rep..

[58] Livak KJ, Schmittgen TD (2001). Analysis of relative gene expression data using real-time quantitative PCR and the 2(-Delta Delta C(T)) Method. Methods.

[59] Schmittgen TD, Livak KJ (2008). Analyzing real-time PCR data by the comparative C(T) method. Nat. Protoc..

