# Supplementary material for: Combined transplantation of human mesenchymal stem cells and human retinal progenitor cells into the subretinal space of RCS rats
Source: Sci Rep. 2017 Mar 15;7:199. doi: 10.1038/s41598-017-00241-5 (PMC5428026; doi:10.1038/s41598-017-00241-5)
Supplement: Supplementary file 1 — Supplementary Information [file 41598_2017_241_MOESM1_ESM.pdf]

Supplementary Information

Combined transplantation of human mesenchymal stem cells and human retinal progenitor cells into the subretinal space of RCS rats

Linghui Qu[1,2a], Lixiong Gao[1,2a], Haiwei Xu[1,2], Ping Duan[1,2], Yuxiao Zeng[1,2], Yong Liu[1,2*], Zheng Qin Yin[1,2*]

1 Southwest Hospital, Southwest Eye Hospital, Third Military Medical University, Chongqing,400038, China

2 Key Lab of Visual Damage and Regeneration & Restoration of Chongqing，Chongqing 400038, China

a These authors contributed equally to this work.

Address correspondence to:

Prof. Yong Liu and Zheng Qin Yin

Email: liu_yong2012@yahoo.com, qinzyin@aliyun.com

Southwest Hospital, Southwest Eye Hospital, Third Military Medical University, Chongqing, China

Tel. +86 23 6875 4401

Supplementary ethical statement

Third Military Medical University of People's Liberation Army

---

Ethical Statement

This is an ethical statement for Lixiong Gao et, al. from Third Military Medical University on the subject of "Combined transplantation of human mesenchymal stem cells and human retinal progenitor cells into the subretinal space of RCS rats" (RCS rats, n = 60). All the related experiment procedures meet the requirements of Laboratory Animal Welfare and Ethics Committee of the Third Military Medical University. The production certificate number for our laboratory animal is SCXK-PLA-20120011, the occupancy permit number is SYXK-PLA-20120031. All the procedures in this study were approved.

Pateat universis per praesentes!

Laboratory Animal Welfare and Ethics Committee

Of the Third Military Medical University

September 18th, 2013

Supplementary figures
Figure 1

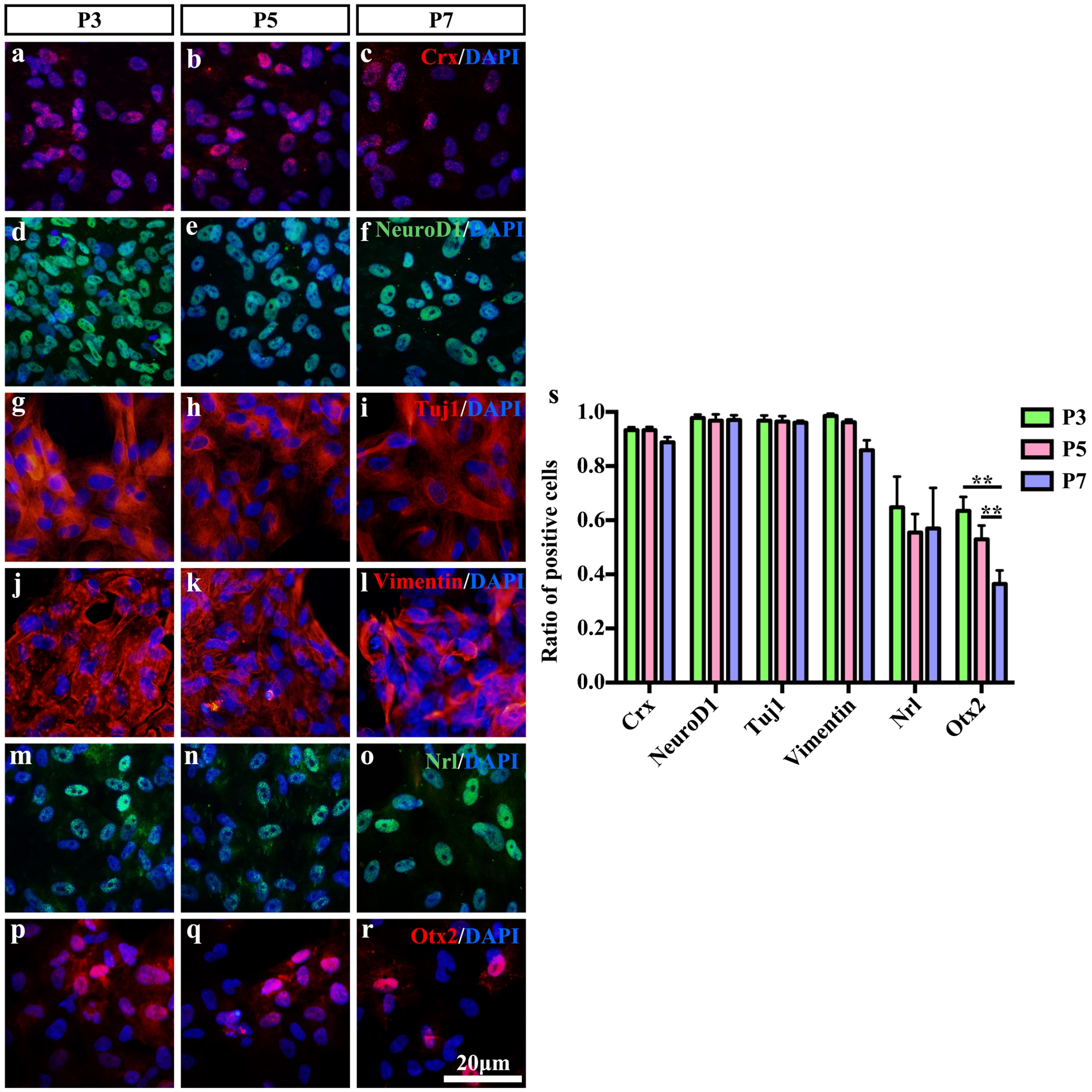

Figure 1 Identification of human retinal progenitor cells (HRPCs). **a-r**: Six markers including Crx, NeuroD1, Tuj1, Vimentin Nrl and Otx2 were used to identify the characteristics of HRPCs in passages3, 5 and 7. **s**: Corresponding statistic analysis of a-r. For Otx2 staining, the significance between P3 and P7 is P = 0.00632. The significance between P5 and P7 is P = 0.00731. Results showed that Crx, NeuroD1, Tuj1, Vimentin and Nrl showed a stable expression ratio during passaging and the expression of Otx2 decreased during passaging . **: P < 0.01.

Figure 2

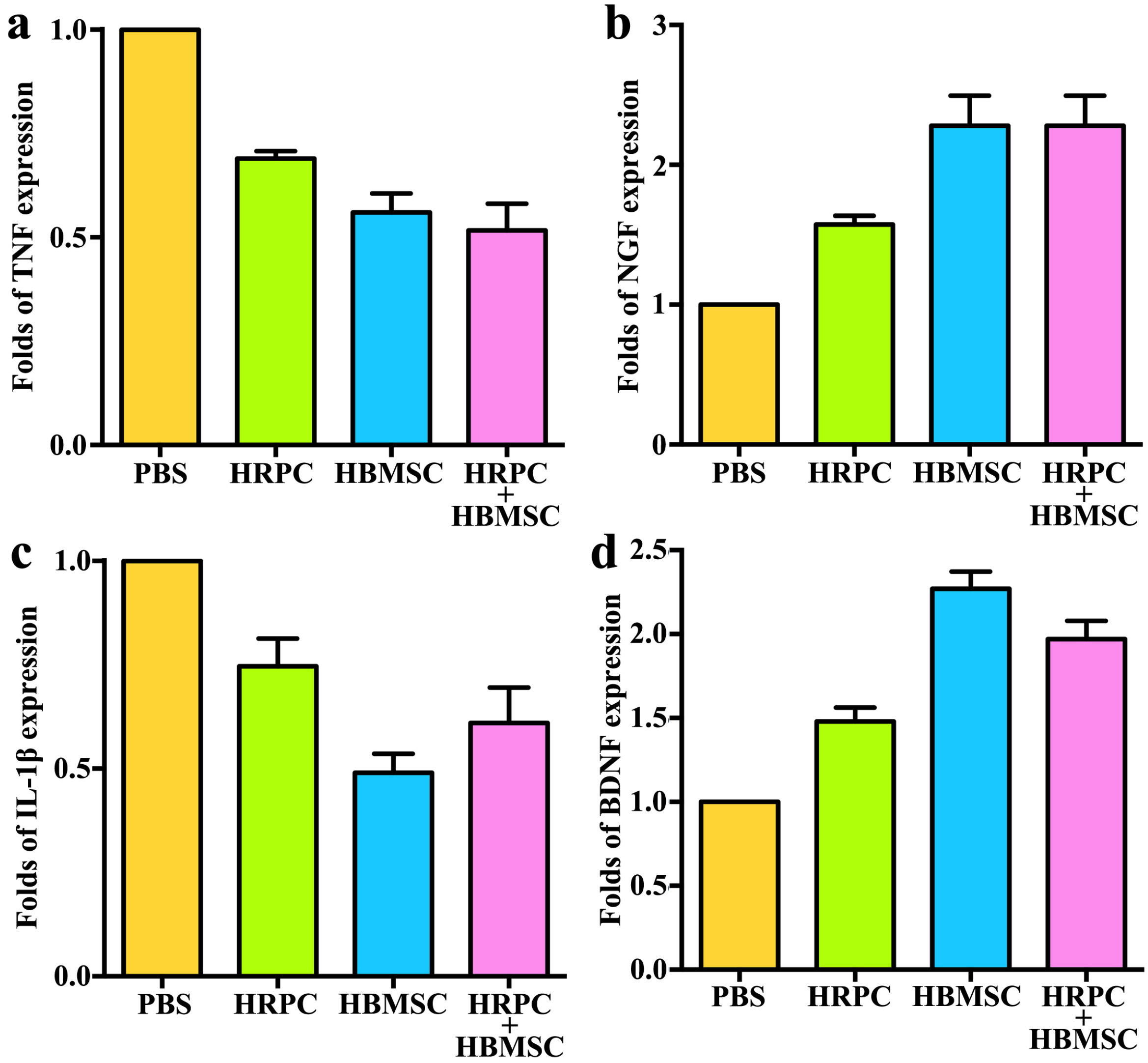

Figure 2 Identification of inflammatory factors and neurotrophic factors after single and combined cell transplantation into subretinal space (SRS). **a**: Folds of TNF expression level. **b**: Folds of NGF expression level. **c**: Folds of IL-1β expression level. **d**: Folds of BDNF expression level.

Figure 3

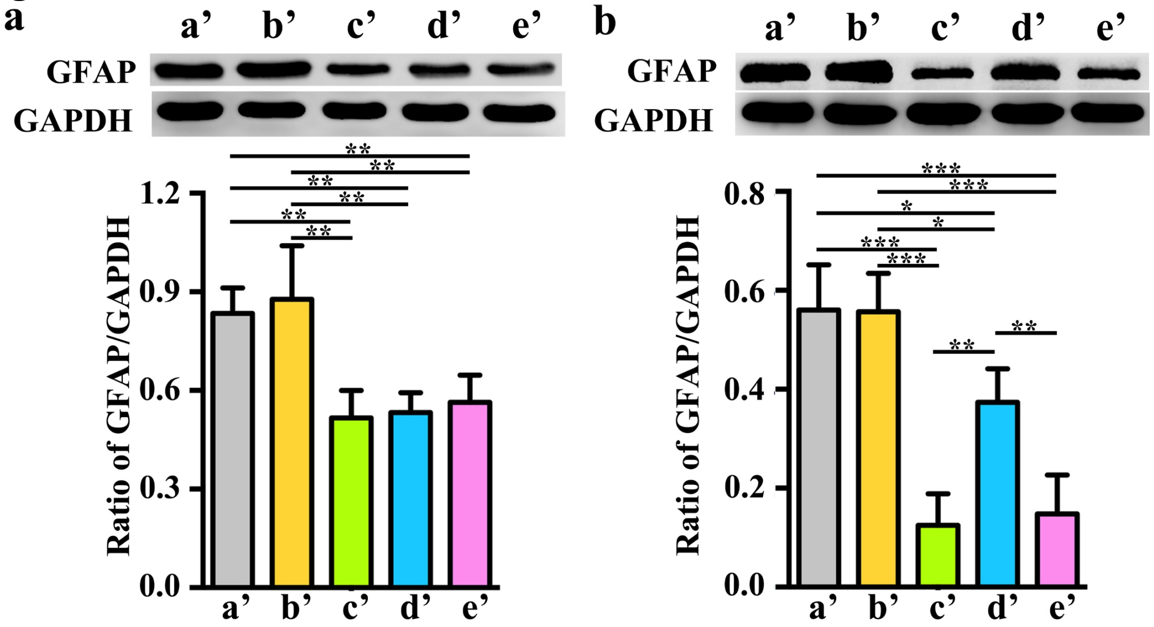

Figure 3 Western blot analysis of the expression of GFAP protein on post operational 6 (**a**) and 12 weeks (**b**) (a': untreated b': PBS; c': HRPCs; d': HBMSCs; e': HRPCs/HBMSCs). Results showed that GFAP expression is significantly suppressed after 6 weeks (a). However, the expression of GFAP in HBMSCs transplantation group is significantly increased after 12 weeks. *: P < 0.05; **: P < 0.01; ***: P < 0.001;

Figure 4

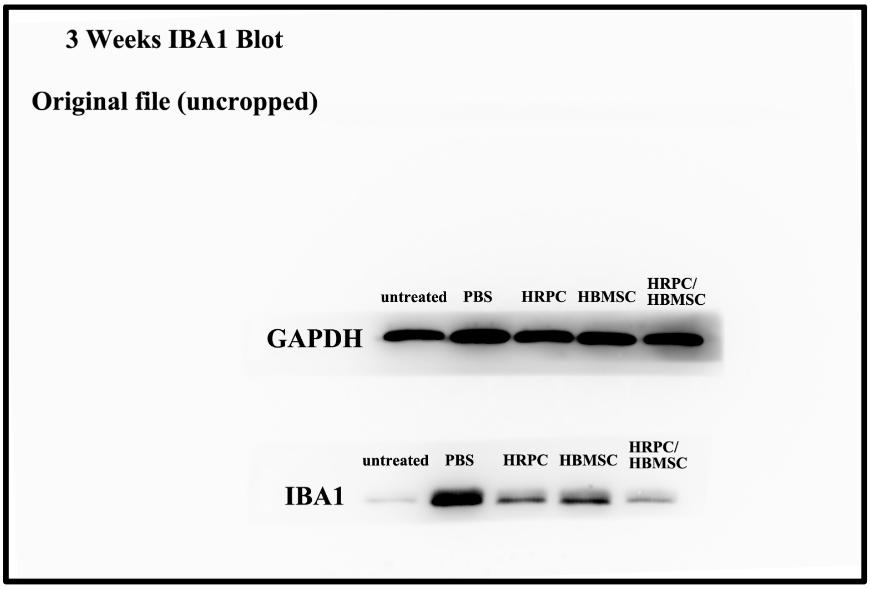

Figure 4 Original file of IBA1 wester-blot at 3 weeks post-operation. The image wasn't cropped.

Figure 5

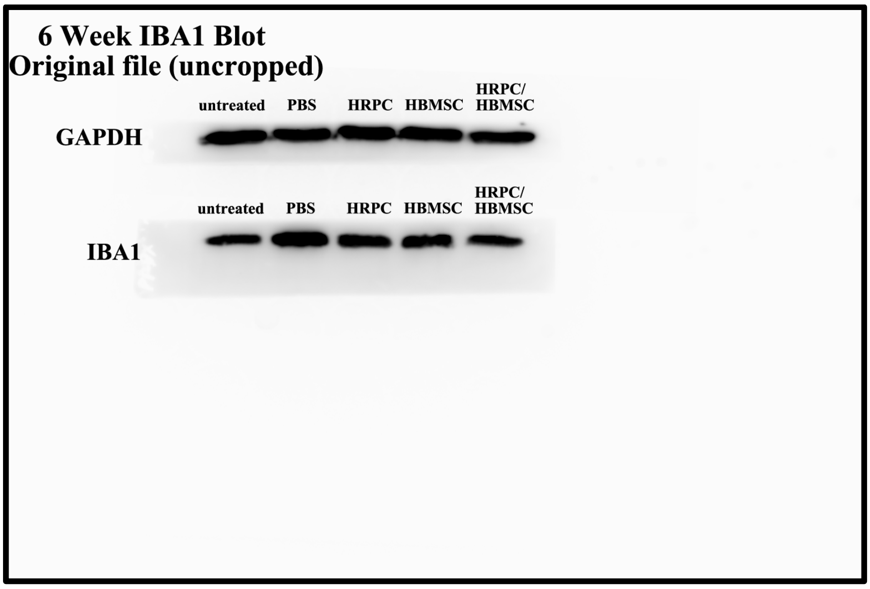

Figure 5 Original file of IBA1 wester-blot at 6 weeks post-operation. The image wasn't cropped.

Supplementary methods

Identification of HRPCs

The immunocytochemistry of HRPCs were performed as previously described1. In brief, after a 15 min fixation with 4% paraformaldehyde (4C°), 10 min penetration with 0.3% Triton X-100 and 15-min blocking with 3% bovine serum albumin, HRPCs plates were then incubated with the primary antibodies including anti-SOX2 (1:500; abcam), anti-Ki67 (1:500; abcam) anti-PAX6 (1:500, abcam) anti-NESTIN (1:800; ermo), anti-OTX2 (1:400, rabbit, abcam), anti-CRX (1:100, rabbit, Santa Cruz Biotechnology), anti-β tubulin Ⅲ (1:500, rabbit, Abcam), anti-Vimentin (1:500, rabbit, Abcam), anti-Nrl (1:100, mouse, Santa Cruz Biotechnology), anti-NeuroD1 (1:400, mouse, Abcam) and anti-GFAP (1:500, rabbit, Sigma Chemical Co) in 1% BSA overnight at 4C°. Cy3-conjugated secondary antibody was then implemented. After counterstaining with 4', 6-diamidino-2-phenylindole (DAPI), HRPCs were viewed and photographed with a confocal microscope.

Freshly purified HRPCs were prepared in cell suspension to perform the FACS test. After washing with Stain Buffer, cells were incubated with FITC- and PE-conjugated primary antibodies, including PAX6 (BD), SOX2 (BD), Nestin (BD) and GFAP (BD) (1 h, 4C°). After washing with wash buffer (Biolegend), HRPCs were then centrifuged at 200 g for 5 min at 4° C. After removing the supernatant, 300 μl 0.01 M PBS was added to re-suspend the cells, which were then transferred to a flow cytometry tube for FACS analysis.

Identification of HBMSCs

FACS was used to identify the characteristics of HBMSCs[2]. In brief, freshly purified HBMSCs were prepared in cell suspension. After washing with Stain Buffer (Biolegend), cells were incubated with FITC-conjugated CD90, PE-conjugated CD44, APC-conjugated CD73 and PerCP-Cy5.5-conjugated CD105 primary antibodies (1 h, 4C°). Simultaneously,

PE-conjugated negative markers, CD11b, CD19/CD34/CD45/HLA-DR, were incubated (1 h, 4C°). After washing with wash buffer (Biolegend), HBMSCs were then centrifuged at 200 g for 5 min at 4°C. After removing the supernatant, cells were resuspended in 300 µl 0.01 M PBS and were then transferred to a flow cytometry tube for FACS analysis (BD Biosciences). After the eighth passage, HMSCs were induced to undergo osteogenic, adipogenic, or chondrogenic differentiation as previously described[3]. For osteogenic differentiation, cells were treated with osteogenic medium for three weeks. Adipogenic stimulation medium consisted of DMEM-LG supplemented with 10% FBS, 50 µg/ml ascorbate-2 phosphate, 10-7 M dexamethasone, and 10 mM β-glycerophosphate (all from Sigma). Cells were cultured for three weeks and were subsequently fixed with 4% formaldehyde and stained with Oil-red O (Sigma). To evaluate the mineralized matrix, differentiated BMSCs were treated with 2% silver nitrate (Sigma) under a UV lamp for 1 h, fixed with 2.5% sodium thiosulfate (Sigma) for 5 min, washed with dH2O, and then counterstained with 1% alizarin red (Sigma). To induce chondrogenesis, cells were cultured as a high-density pellet (2.5 × 105 cells/pellet) in DMEM-LG serum-free medium supplemented with 10-7 M dexamethasone, 40 µg/ml L-proline, 100 µg/ml sodium pyruvate, 1% ITS-premix, and 10 ng/ml transforming growth factor-β3 (TGF-β3) (R&D Systems, Minneapolis, MN) for four weeks. The extracellular matrix in the chondrocyte-like beads and the pellets were embedded in paraffin, cut into histological sections and stained with alcian blue (Sigma).

Supplementary Table

Summary of primers.

Genes	Primers	Sequences
BDNF	Forward	ctctttctgctggaggaataca
	Reverse	ctgtgacccactcgctaatac
TNF-α	Forward	cccctttatcgtctactcctca
	Reverse	gccactacttcagcgtctcg
IL-1β	Forward	ctgtgactcgtgggatgatgac
	Reverse	ggagaataccacttgttggctta
GAPDH	Forward	agacagccgcatcttcttgt
	Reverse	tgatggcaacaatgtccact

Reference:

1 Hamel, C. Retinitis pigmentosa. *Orphanet. J. Rare Dis.* **1**, 40 (2006).

2 Schwartz, S. D. *et al.* Embryonic stem cell trials for macular degeneration: a preliminary report. *Lancet* **379**, 713-720 (2012).

3 Duan, P., Xu, H., Zeng, Y., Wang, Y. & Yin, Z. Q. Human bone marrow stromal cells can differentiate to a retinal pigment epithelial phenotype when co-cultured with pig retinal pigment epithelium using a transwell system. *Cell Physiol. Biochem.* **31**, 601-613 (2013).
